# Sex Differences of Cardiolipin in Tissue Distribution Based on Targeted Lipidomic Analysis by UHPLC-QTOF-MS/MS

**DOI:** 10.3390/molecules27206988

**Published:** 2022-10-18

**Authors:** Yuqi Lin, Xugui Li, Mengxiang Dai, Qiaoyu Li, Qingxin Shi, Lijun Zhang, Rongzeng Huang, Chengwu Song, Shuna Jin

**Affiliations:** 1 College of Pharmacy, Hubei University of Chinese Medicine, Wuhan 430065, China; 2 Hubei 672 Orthopaedics Hospital of Integrated Chinese and Western Medicine, Wuhan 430079, China; 3 College of Basic Medicine, Hubei University of Chinese Medicine, Wuhan 430065, China; 4 Key Laboratory of Traditional Chinese Medicine Resources and Chemistry of Hubei Province, Wuhan 430065, China

**Keywords:** cardiolipin, LC-MS/MS, tissue distribution, sex difference, targeted lipidomics, multivariate statistical analysis

## Abstract

Cardiolipins (CLs) are involved in ATP production, mitochondria biogenesis, apoptosis and mitophagy. Their tissue distribution can provide insight into the function of mitochondria and related diseases. However, the reports on tissue distribution of CLs remain limited. In this research, CLs were identified from heart, liver, kidney, spleen, lung, skeletal muscle, and brain using ultra-high-performance liquid chromatography coupled with quadrupole time-of-flight mass spectrometry (UHPLC-QTOF-MS/MS). Then, the distribution and sex difference of CLs in seven tissues were compared by a targeted lipidomic approach. A total of 88 CLs were identified, of which 58, 51, 57, 58, 50, 61 and 52 CLs were found in heart, liver, kidney, spleen, lung, skeletal muscle, and brain, respectively. Compared with the distribution of CLs in heart, liver, kidney, and skeletal muscle, the CLs in spleen, lung, and brain showed significant differences. Moreover, the results indicated that there were sex differences of CLs in liver and kidney. A total of 16 CLs in liver tissue and 21 CLs in kidney tissue, with significant sex differences, were screened. Our findings in the targeted lipidomic analysis demonstrated that tissue distribution of CLs was essential in the dynamic states and sex differences of CLs, which might provide evidence for the mitochondrial-related mechanism under physiological and pathological conditions.

## 1. Introduction

Cardiolipins (CLs) are anionic phospholipids, which mainly exist in the mitochondria. They play an important role in mitochondrial energy metabolism. Especially in the inner membrane of mitochondria, CLs can act as a functional “glue” that provides efficient transfer of electrons and protons through components connected to the mitochondrial respiratory chain [1]. In addition, CLs play leading roles in eliciting mitochondria-mediated cell apoptosis and mitochondria-specific autophagy. CLs have also been reported to be involved in mitochondrial protein importation, maintaining membrane fluidity and the activity of cytochrome C [2]. They often migrate to the outer membrane of the mitochondria when the mitochondrial membrane is damaged [3].

The role of CLs in a variety of pathological processes has accumulated in a wealth of knowledge [4,5]. Peroxidation, loss of CL content, and impaired acyl chain remodeling are related to certain disease states, including diabetic cardiomyopathy [6], Barth Syndrome (BTHS) [7], heart failure [8] and neurological diseases [9]. In addition, accumulating evidence has suggested that the altered CLs can be biomarkers or predictors to reflect the state of diseases. Previous studies showed that the total content of CL decreased and the predominant fatty acyl species of L4-CL (18:2) changed, to longer polyunsaturated fatty acids, in the type II diabetic mouse [7,10]. In BTHS, tetralinoleyl-CL concentrations were reported to show a specific decrease, while monolysocardiolipin (MLCL) increased, indicating that the ratio of MLCL/CL can be used as a biomarker of BTHS [11]. Grudis et al. found that the expression of MLCL acyltransferase and tafazzin reduced by 66 and 43%, respectively, in failing idiopathic dilated cardiomyopathic (IDC) tissue, compared to non-failing control, which suggests that changes in CL remodeling and biosynthesis could play a key role in heart failure progression [12]. There is evidence that loss of CL content effects mitochondrial synaptic dysfunction and oxidative stress in Alzheimer’s disease (AD) [13]. Furthermore, it was reported that the spatial distribution of CL in oncocytic thyroid tissues overlapped with the accumulation of mitochondria-rich oncocytic tumor cells [14]. Since disorders of the CL metabolism are closely related to many pathological consequences, the comprehensive investigation of CL in different tissues would contribute towards discovering its potential role.

Liquid chromatography tandem mass spectrometry (LC-MS) is a key technique for analysis of CL, due to its reduced ion-suppression effects, separation and detection of isomers and isobars, and the possibility of separating compounds based on their physicochemical properties [15,16]. In a previous study, the shotgun strategy was also used to directly analyze CL by intra-source separation without chromatographic separation. However, complete isolation and quantification of low-abundance compounds that present in the entire CL class cannot be achieved [17]. Analysis of CL has been mainly focused on two analytical strategies: untargeted and targeted lipidomics [18]. The strategy of the non-targeted analysis uses computational sorting or multivariate pattern-recognition analysis to classify all the detectable metabolites. Thus, the method does not require prior knowledge of the analytes. The targeted strategy is applied to analyze lipids with known fragmentation patterns. CL, as a class of essential phospholipids, has a general structural relationship and similar core structure. Thus, the molecular formula and major characteristic fragments of each CL can be predicted. Ultra-high performance liquid chromatography coupled with quadrupole time-of-flight mass spectrometry (UHPLC-QTOF-MS/MS) combines the sensitivity and selectivity of QQQ instruments with TOF technology, providing high resolution and mass accuracy. Therefore, UHPLC-QTOF-MS/MS is a suitable analytical technique with which to analysis CL based on the targeted lipidomic. In this method, multivariate statistical techniques were performed on the high-dimensional data sets acquired by UPLC-QTOF-MS/MS. The information of data could be quickly read through the mathematical models. The strategy has been proven to be an efficient tool for comprehensive analysis of CL in a large number of tissue samples [19].

In the present study, CL was identified and characterized in the heart, liver, kidney, spleen, lung, brain and skeletal muscle of rats, by UHPLC-QTOF-MS/MS. The distribution of CL in these 7 tissues was compared based on the targeted lipidomic strategy. Through the multivariate statistical method, sex differences of CL in the tissues were further investigated. The final screened CLs with significant sex differences may contribute towards exploring its potential role in metabolic diseases and explaining the mechanism linked to sex differences.

## 2. Results

### 2.1. MS/MS Fragmentation Patterns of CLs

**Figure 1:**
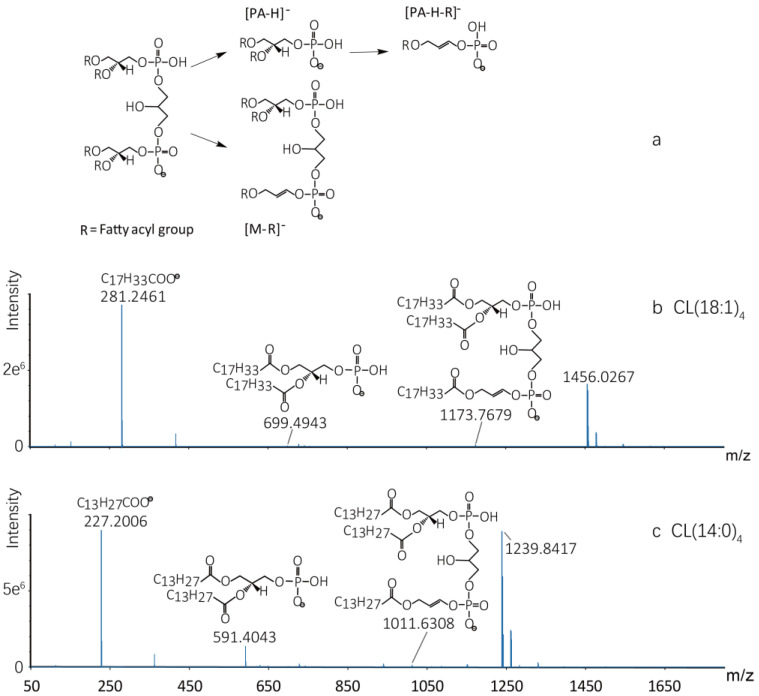
The chemical structure and characteristic product ions of CLs. (**a**) The chemical structures of CLs, consisting of two phospholipid groups, three glycerol groups and four fatty acyl chains. [PA-H]^−^, [PA-H-R]^−^ and [M-R]^−^ are the major characteristic fragments. (**b**) The chemical structures and characteristic product ions of CL (18:1)_4_. (**c**) The chemical structures and characteristic product ions of CL (14:0)_4_.

CLs usually consist of two phospholipid groups, three glycerol groups and four fatty acyl chains. As depicted in Figure 1a, they had similar MS behavior, resulting in a series of regular and predictable product ions. The major characteristic fragments of CLs were [PA-H]^−^, fatty acyl and [M-R]^−^ in negative mode. Two standards of CL (18:1)_4_ and CL (14:0)_4_ were characterized and detected primarily as [M-H]^−^ ions prior to the analysis of the CL molecular species in rats. As shown in Figure 1b, the most sensitive diagnostic product ions of CL (18:1)_4_ were the product ions at *m/z* 669.4943, 1173.7679 and 281.2461. Similar to CL (18:1)_4_, the fragmentation patterns of CL (14:0)_4_ is depicted in Figure 1c. The most sensitive diagnostic product ions of CL (14:0)_4_ were at *m/z* 591.4043, 1011.6308 and 227.2006. In this research, the product ion [PA-H]^−^, fatty acyl and [M-R]^−^ were used as diagnostic ions for targeted identification and screening of CLs among seven tissues.

### 2.2. Identification of CLs in Tissues

In the data mining step, 262 CLs were summarized from cells, animals and plants, according to former references [20,21,22,23,24]. Based on the fragmentation patterns of CLs with similar product ions, the possible product ions had been derived, as shown above. The molecular ion peaks of target CLs were manually matched by the charge-mass ratio. Then, whether the chromatographic behavior of product ions and precursor ions were present and consistent was checked. Unreasonable ions were excluded during the process of matching. The charge–mass ratio and retention time of the identified compounds were determined. 

The combination of four fatty acid groups led to an extremely complicated CL species. Therefore, isotopic peaks of some CLs were easily confused with many other CLs of low abundance. False-positive identities must be excluded from the MS data. In the present research, the isotopic abundance ratio of CL could be calculated and thus be used to exclude the relative isotopic molecular ion peaks. For instance, one peak at the retention time 9.72 min, was detected in the EIC at *m/z* 1449.9779. A corresponding signal of *m/z* 1451.9902 was identified as the isotopic peak by the evidence of its same retention time and the peak intensity ratio to *m/z* 1449.9779 (see Supplementary Materials; Figure S1). For excluding isotopic false-positive identities, all CLs identified in this research were checked one by one, using the above method.

Furthermore, some false-positive peaks with molecular ions at [M-H]^−^ but without [M-2H+Na]^−^ were also discovered, due to the in-source fragmentation and background noise [25]. These identified CLs were further checked with molecular ions at [M-2H]^2−^. The peaks at [M−2H]^2−^ provided evidence to support the identification at the same retention time. Thus, the peaks were proven to be a false positive.

**Figure 2:**
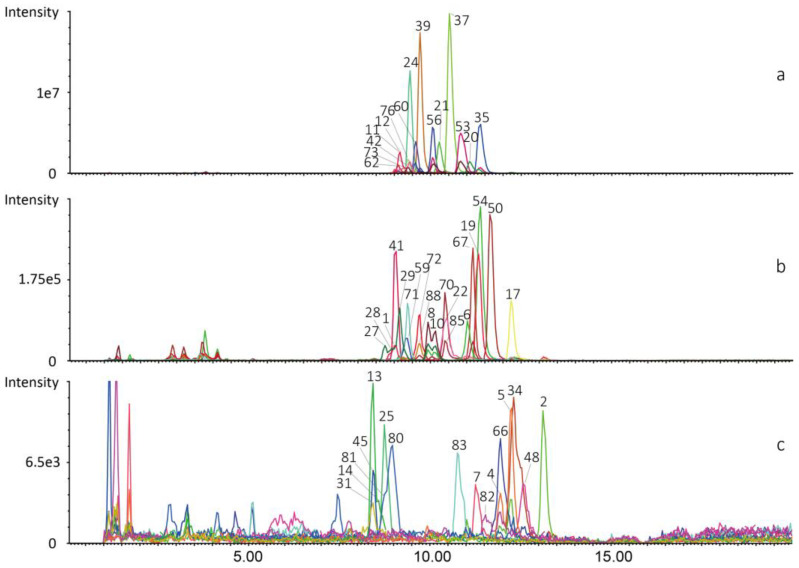
Integration of individual peak in extracted ion chromatograms (EICs) of the 58 CLs in liver tissue by UPLC-QTOF-MS/MS. (**a**) CLs with peak intensity >1.93 × 10^5^ and <1.2 × 10^7^ cps. (**b**) CLs with peak intensity >1.54 × 10^4^ and <2 × 10^5^ cps. (**c**) CLs with peak intensity >1.46 × 10^3^ and <8.3 × 10^3^ cps. Peak numbers of compounds are consistent with those in Table S1.

Finally, the targeted profiles of CLs were established by integration of the 88 CLs in seven tissues (58 in heart, 51 in liver, 57 in kidney, 58 in spleen, 50 in lung, 61 in skeletal muscle and 52 in brain). The LC-MS/MS information for identified CLs in all tissue samples is shown in Table S1. The representative profile of CLs in the liver sample is depicted in Figure 2.

### 2.3. CL Distribution in Seven Tissues Based on Targeted Lipidomic Analysis

**Figure 3:**
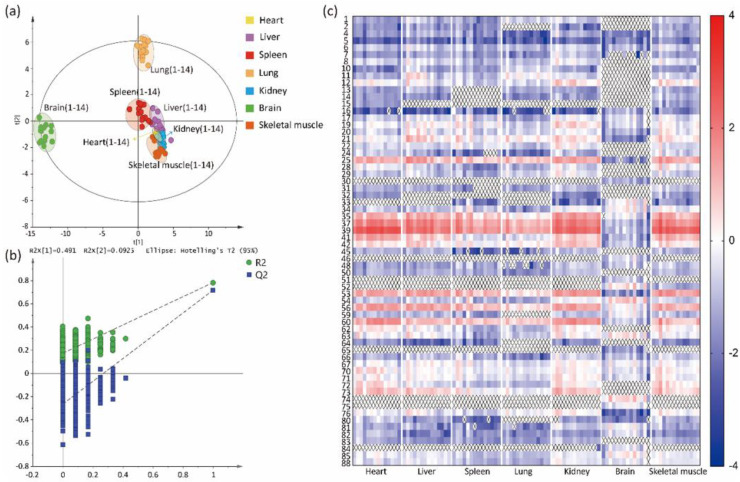
(**a**) OPLS-DA scores scatter plot of heart, liver, spleen, lung, kidney, brain, and skeletal muscle. (**b**) The 999 permutation tests for the OPLS-DA models based on seven tissues. (**c**) Changed multiple of the relative contents of 64 CLs, detected in seven tissues. Quantitation of CLs was performed by dividing the peak area of each CL by the peak area of CL (14:0)_4_. The value of each compound was the log_10_ transformation of the relative content. The CLs not detected in the seven tissues were marked with x.

The distribution of CLs in 7 tissues, from 14 rats (7 MM and 7 FF), was compared by targeted lipidomic analysis. In the PCA model, there was a separation between brain and other tissues, which indicated a difference in the content of CLs (see Figure S2). R^2^X and Q^2^ were 0.865 and 0.639, respectively. R^2^X is used to measure how well the model fits the data. A large R^2^X (close to 1) is a necessary condition for a good model, but it is not sufficient. Q^2^ indicates how well the model predicts new data. A large Q^2^ (Q^2^ > 0.5) indicates good predictivity. To better study how the other groups were separated, PCA was performed after excluding the brain samples. The results indicated that the spleen and lung group separated well (see Figure S3). The supervised OPLS-DA was used to further explore the differences in seven tissues. In OPLS-DA analysis, the trend of CL separation in seven tissues was similar to PCA, as shown in Figure 3a. The CLs in heart, liver, kidney, and skeletal muscle converged. Thus, the results indicated that the distribution of CLs in brain, spleen and lung differed from the CLs in heart, liver, kidney and skeletal muscle. The R^2^Y and Q^2^ of OPLS-DA models were 0.854 and 0.796, respectively, which suggested that the models had good abilities for prediction and reliability. Furthermore, the Q^2^ of the 999-time permutation tests was −0.264 (see Figure 3b), indicating that the OPLS-DA models were not overfitting.

To improve the visualization, CL profiles of 7 tissues were displayed in a heatmap. As illustrated in Figure 3c (data were shown in Table S2), distribution of the 64 compounds in the 7 tissues were analyzed. The color intensity, from blue (−4) to red (3), reflected the relative content of each CL. It was obvious that there were differences, in the CLs, between the brain and the other six tissues. In heart, liver, kidney and skeletal muscle, the distribution of relative CL content was similar. Further, more detailed differentiations of CLs in the seven tissues were analyzed. The CLs, based on the number of C atoms, were divided into seven groups: C66 group, C68 group, C70 group, C72 group, C74 group, C76 group, and C78 group. As shown in Table 1, each group of CLs was different in the seven tissues. The relative contents of total CLs were the highest in heart, followed by skeletal muscle, kidney, liver, spleen, lung and brain. However, the relative contents of the C78 group were the highest in brain tissue and the relative contents of the C68, C74, and C76 groups were the highest in kidney tissue. In addition, there were no CLs of the C66 group in brain tissue.

### 2.4. Sex Differences of CLs in Tissues

**Figure 4:**
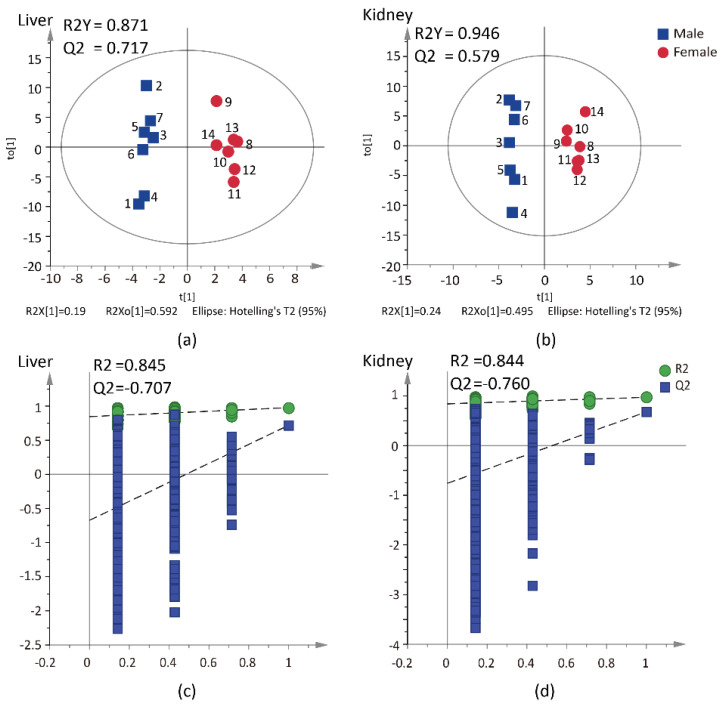
The results of OPLS-DA models and 999 permutation tests based on male and female in liver and kidney tissues. (**a**) The score plots from OPLS-DA model of liver. (**b**) The score plots from OPLS-DA model of kidney. (**c**) The validation plots from 999-permutation test of liver. (**d**) The validation plots from 999-permutation test of kidney.

PCA and OPLS-DA were further used for examining the differences of CLs between the male and female groups, in seven tissues. Table 2 shows the R^2^ and Q^2^ values of seven tissues. The Q^2^ value of OPLS-DA indicates that the models of heart, lung, brain and skeletal muscle displayed poor reliability. Moreover, the 999-time permutation tests showed that the spleen models were overfitting. To verify the above results, a Wilcoxon–Mann–Whitney test was conducted, to compare the relative contents of the CLs in the 7 tissues. There were 2, 4, 1 and 8 CLs with sex differences in heart, spleen, lung and skeletal muscle, respectively, while there were 16 and 21 CLs with sex differences in liver and kidney, respectively. Moreover, the results of PCA-X models suggested that CLs of the male and female groups were separated well, both in liver and kidney (see Figure S3), which indicated sex differences in the levels of CLs. Then, the OPLS-DA was used to further explore the sex differences. As Figure 4 shows, in OPLS-DA models, the R^2^Y and Q^2^ values in liver were 0.871 and 0.717, respectively, and the kidney values were 0.946 and 0.579, respectively, which also indicated that the two models had good abilities for prediction and reliability. Meanwhile, 999-permutation tests were conducted to test the validity and predictability of two OPLS-DA models. R^2^ and Q^2^ represent goodness of fit of the model. The criteria for validity are that the Q^2^-points intersect the vertical axis (on the left) at, or below zero. The results showed that Q^2^ were both negative and the two OPLS-DA models were not overfitting (see Figure 4c,d).

**Figure 5:**
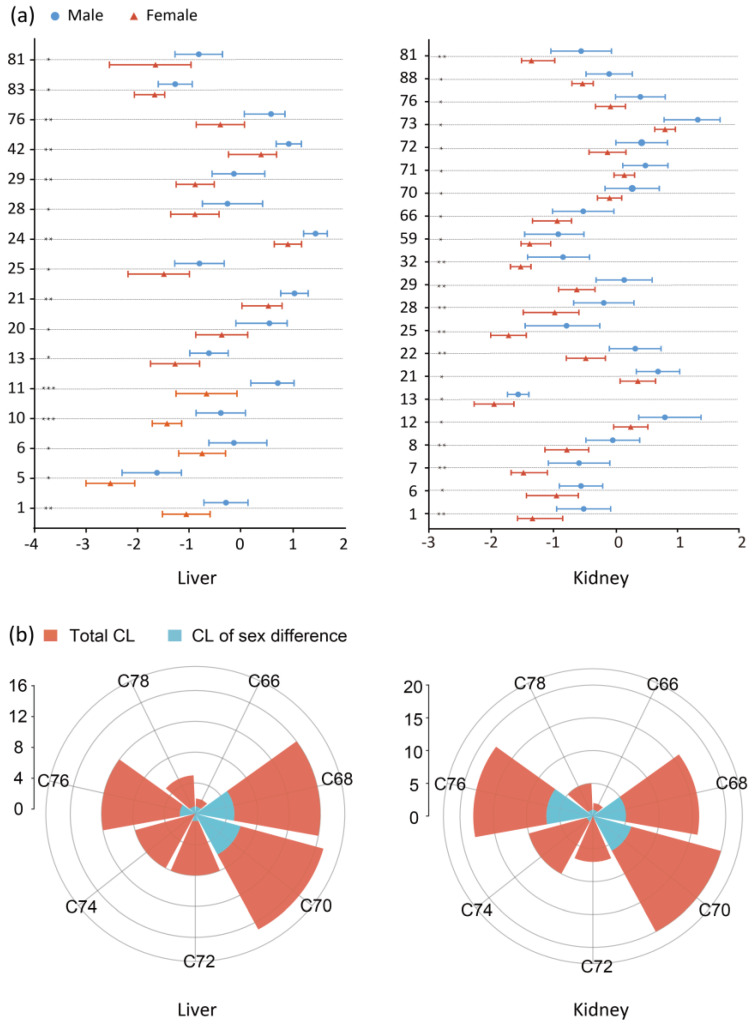
(**a**) Relative contents of CLs detected in different sex of liver and kidney tissues. The results were presented as the mean ± SD (n = 7). * *p* < 0.05, ** *p* < 0.01, *** *p* < 0.001 for male vs. female. The values of each CL were the log_10_ logarithm transformation of their relative peak area. The numbers of compounds are consistent with those in Table 1. (**b**) The distributions of significant differential CLs and total CLs in tissues, stratified by the C number in liver and kidney tissues. The CLs based on the number of C atoms were divided into seven groups. The diameter is equal to the number of CLs.

According to the OPLS-DA analysis (VIP > 1.0) and nonparametric test (*p* value < 0.05), there were 16 CLs and 21 CLs with significant sex differences, in liver and kidney, respectively. The CLs with significant sex differences in liver and kidney were illustrated as the error line diagram of fold changes (see Figure 5a). The fold change of a CL was the mean relative level of a CL in the male and female groups. It was obvious that in both liver and kidney tissues, all CLs with significant sex differences in female rats were lower than those in male rats. Meanwhile, the contents of all CLs in liver and kidney were compared between male and female rats, as shown in Table 3. The results also suggested that the contents in all 7 CL groups, in female rats (groups C66, C68, C70, C72, C74, C76 and C78), were lower than that in male rats.

The distribution of CLs with sex difference were further investigated in each group, classified by the number of C atoms. Figure 5b shows the distributions of CLs, with sex difference, in liver and kidney. In liver, among the 16 CLs displaying significant sex differences, the proportions of CLs with C68 and C70 were higher, at 45% and 54%, respectively. In kidney, groups C68, C70 and C76 showed a higher proportion of CLs with sex differences, which were 45%, 42% and 63%, respectively.

## 3. Discussion

In this study, the distribution and sex differences of CLs in seven tissues were analyzed using targeted lipidomic analysis by UHPLC-QTOF-MS/MS. As a result, the CL profiles of seven tissues in rats were established, by integration of 88 CLs (58 in heart, 51 in liver, 57 in kidney, 58 in spleen, 50 in lung, 61 in skeletal muscle, and 52 in brain). There were differences in the distribution of CLs in the seven tissues. Moreover, comparing the relative contents of CLs in liver and kidney tissues between males and females, suggested significant sex differences. Further results showed that sex differences in liver tissue occurred in CLs with C68 and C70, and those in kidney tissue occurred in CLs with C68, C70 and C76.

Our findings indicated that the content of most CLs was higher in skeletal muscle, heart, kidney and liver, while the content of CL was lower, and different, in lung, spleen, and brain. The CLs’ metabolic profile in seven tissues reflected the different metabolic roles. The results were consistent with the previous studies, which had pointed out that CL levels were strongly related to the oxidative metabolism [26,27]. In tissues with periodic oxidative activity, the content of CL was similar, such as skeletal muscle, heart, kidney and liver. The low level of most CLs in brain could be determined by the fact that the energy requirement per mass unit in the brain is almost half that of the heart or kidney [28]. At the same time, previous reports demonstrated that the concentration of linoleic acid (18:2n−6) were higher in the heart than in the liver, lung, spleen, skeletal muscle and kidney [29], which also agreed with our results that the content of C72 (containing linoleic acid) was higher in the heart than in other tissues. Moreover, CL species were different in the same tissue. In the lung tissue, compounds 45 and 48 were present in some samples and not in others. The reason may be that rats have individual differences [30].

In addition, the results indicated that CLs in liver and kidney had sex differences and CL contents in females were lower than those in males. Reproduction has introduced the flexibility necessary to tailor metabolism according to reproductive status in females’ liver, consequently diverging from that in males [31]. Thus, energy metabolism has gender specificity in liver [31]. Sex differences in kidney were evident in testosterone receptors and estrogen receptors [32]. There is sufficient evidence that CLs are a class of functional phospholipids in the inner membrane of mitochondria [33]. Thus, the difference in CLs is significantly associated with the sex dimorphism of mitochondria in liver and kidney [34,35]. Mitochondria belonging exclusively to maternal transmission exerted differential effects in males and females [36]. It is also reported that, compared to males, females have shown lower mitochondrial content [36]. However, female mitochondria have indicated more specific activity and less oxidative damage, in contrast to males [37]. In addition, sex differences compared against the effects of sex hormones on CL synthesis or remodeling have been well established, and the results suggest that enzymes are more abundantly transcribed in males than females [38]. Although there is evidence for potential mechanisms underlying the sex differences in CL, future functional studies are needed to confirm the observed associations.

The screened CLs in the study might be representative components for sex differences in liver and kidney. As reported, the prevalence of non-alcoholic fatty liver disease (NAFLD) and non-alcoholic steatohepatitis (NASH) vary, due to different responses in male and female, which are often accompanied by mitochondrial dysfunction [39]. CLs have been found to be able to affect mitochondrial biogenesis, morphology, fusion and fission, respiration, and protein import [40]. Therefore, the CLs with sex differences might also be a potential mechanism to NAFLD or NASH. Moreover, chronic kidney diseases (CKD) such as kidney failure and diabetic kidney disease (DKD) are reported to be associated with mitochondrial dysfunction [41,42]. In addition, lipid metabolism is often altered in patients with CKD, leading to the progression of kidney damage [43]. Especially in DKD, the cardinal sign of disrupted mitochondrial architecture and bioenergetics is abnormal cardiolipin [44,45]. Regulating cardiolipin remodeling and reducing cardiolipin peroxidation could reverted DKD progression [46,47]. Therefore, studies aimed at the content and function of targeting CLs might be beneficial for the treatment of some mitochondrial-related diseases. 

## 4. Materials and Methods

### 4.1. Chemicals and Materials

Acetonitrile was obtained from Thermo Fisher Scientific (Waltham, MA, USA). Ammonium formate (AF) was obtained from Sinopharm Chemical Reagent Co., Ltd. (Shanghai, China). 2-propanol, chloroform, and methanol were purchased from Sigma Chemical Co., Ltd. (St. Louis, MO, USA). The Milli-Q water system was used to produce deionized water in this study (Millipore, Bedford, MA, USA). The reference standards of CL (18:1)_4_ and CL (14:0)_4_ were obtained from Avanti Polar Lipids Inc. (Alabaster, AL, USA). 

### 4.2. Sample Preparation

Appropriate amounts of CL (18:1)_4_ and CL (14:0)_4_ were separately dissolved in acetonitrile/2-propanol (1:1, *v*/*v*) to prepare 200 μg/mL stock solution. The working solution was diluted with acetonitrile/2-propanol (1:1, *v*/*v*) to obtain the concentration of 10 μg/mL. Seven female and seven male SD rats (200–220 g) were obtained from Hubei Province Center for Disease Control and Prevention (Wuhan, China), with certification SCXK 2021-0011. All rats were acclimatized for one week. Then, each rat was dissected quickly after anesthetization with pentobarbital injection. Tissues, including heart, liver, kidney, spleen, lung, skeletal muscle, and brain were removed and immersed in ice-cold saline. All tissues were stripped of blood and fat. The Animal Ethics Committee of Hubei University of Chinese Medicine had approved all animal procedures. The method of Bligh and Dyer was used to extract lipids [17,48]. A 50 mg sample from each tissue and 200 μL CL (14:0)_4_ of 500 ng/mL (internal standard) were added to the tissue grinder and mixed with 1 mL chloroform and 1 mL methanol to grind to a homogenate, then transferred to a test tube. Further, 1 mL chloroform, 1 mL methanol, and 1.8 mL 0.9% NaCl were then added to each test tube. After centrifugation at 4000 rpm, the chloroform layer of the sample was removed and stored. Then, 2 mL of chloroform was added to the MeOH/H_2_O layer, again. The sample was then centrifuged, as above. The chloroform layer was combined and dried under a nitrogen dryer. Finally, each lipid extract was reconstituted in 200 μL of acetonitrile/2-propanol (1:1, *v*/*v*).

### 4.3. LC/MS Conditions

The ACQUITY UPLC M−Class system was used for UHPLC-MS/MS analysis. A Waters ACQUITY UPLC BEH C18 column (100 × 2.1 mm, 1.7 μm) was used for separation. The injection volume was 5.0 μL and the flow rate was 0.3 mL·min^−1^. The solvent conditions were optimized. As a result, the mobile phase A and the mobile phase B were 5 mM ammonium formate water and 5 mM ammonium formate in methanol/2-propanol (1:1, *v*/*v*), respectively. The binary gradient used for the 20-min chromatogram was set: 90% B for 0.1 min, 90% B to 95% B for 6 min, 95% B to 98% B for 9 min, hold 98% B for 3 min, 98% B to 90% B for 0.1 min and maintain at 90% B for 1.9 min.

The Waters Xevo G2-XS QTof mass spectrometer was operated in the electrospray ionization (ESI) mode (Waters, Milford, MA, USA). The negative ion electrospray was selected for data acquisition. The optimized operating parameters were set: cone gas flow, 50 L·h^−1^; capillary voltage, 2.5 kV; source temperature, 100 °C; cone voltage, 40 V; desolvation temperature, 500 °C; desolvation gas flow, 500 L·h^−1^. The mass ranges were set at *m/z* 50–1800 for full scan, with scan duration of 1 s. The MSE continuum mode was used to collect data.

### 4.4. Data Processing

Acquirement and analysis of LC-MS/MS data were achieved by using the LC-QTOF-MS/MS and MassLynx 4.1 software (Waters, Milford, MA, USA), respectively. Quantitation of CLs was performed by dividing the peak area of each CL by the peak area of CL (14:0)_4_. GraphPad Prism 7 (GraphPad Software Inc., San Diego, CA, USA) and SIMCA-P (v13.0, Umetrics, Umeå, Sweden) were used to conduct statistical analyses. The results were presented as the means ± SD.

During multivariate statistical analysis, the data was converted logarithmically (log_10_ transformation) and the missing value was set as −9. The whole data was analyzed with UV scaling. Tissues were set as groups: heart, liver, kidney, spleen, lung, skeletal muscle, and brain, to evaluate the differentiation of tissue distribution. First, principal component analysis (PCA) and orthogonal partial least squares discrimination analyses (OPLS-DA) were used to explore the clusters and trends of CLs among seven tissues. Then, PCA and OPLS-DA were also used to explore the clusters and trends of CLs among the male and female in each tissue, to further assess the differentiation of CLs caused by sex difference. Therefore, permutation tests (999 cycles) were performed to evaluate model overfitting. For assessing the goodness-of-fit and predictive ability of OPLS-DA model, the R^2^Y and Q^2^ (cross-validation parameters) were calculated. The parameter of variable importance in the projection (VIP) showed the significance of a variable in a model. In OPLS-DA model, a VIP value greater than 1.0 was one of the criteria for screening differential CLs between female and male tissue samples, while the other criterion was that the contents of CLs showed significant differences (*p* value < 0.05).

## 5. Conclusions

In conclusion, the CL profile was established using UHPLC-QTOF-MS/MS combined with targeted lipidomic, and a total of 88 CLs were characterized in seven tissues. The established metabolite profile can facilitate the qualitative and relative quantitative analysis of CLs in tissues. The sex-specific CLs in liver and kidney can be used as an indicator for explaining the differences between males and females. The present study has shown that the targeted lipidomic analysis is an effective tool for differentiating tissue distribution and sex-specific differences in tissues. Further studies will focus on the mechanisms involved in CL function and sex differences.

## Figures and Tables

**Table 1:** The relative content of C66 group, C68 group, C70 group, C72 group, C74 group, C76 group and C78 group in seven tissues.

CLs	Heart	Liver	Spleen	Lung	Kidney	Brain	Skeletal Muscle
C66	0.04 ± 0.03	0.29 ± 0.44	0.05 ± 0.06	0.32 ± 0.39	0.32 ± 0.36	0.00 ± 0.00	0.15 ± 0.18
C68	2.14 ± 1.57	5.57 ± 7.36	1.28 ± 1.52	3.09 ± 2.96	7.36 ± 7.46	0.17 ± 0.18	5.11 ± 5.95
C70	20.70 ± 12.41	40.39 ± 41.43	16.35 ± 19.25	12.28 ± 11.83	38.98 ± 26.03	1.05 ± 1.10	33.01 ± 33.26
C72	649.90 ± 275.46	264.69 ± 186.12	101.10 ± 106.54	44.70 ± 41.02	375.99 ± 191.13	5.20 ± 6.05	437.38 ± 334.38
C74	102.71 ± 51.97	68.57 ± 40.61	35.86 ± 33.52	13.51 ± 9.92	115.12 ± 55.49	15.76 ± 17.60	70.20 ± 71.39
C76	20.27 ± 12.86	12.64 ± 10.86	6.27 ± 7.35	3.41 ± 3.52	21.51 ± 17.40	8.58 ± 9.49	17.17 ± 21.70
C78	1.60 ± 1.46	1.11 ± 0.66	0.43 ± 0.48	0.30 ± 0.29	1.51 ± 1.14	3.56 ± 3.91	1.10 ± 0.95
All	797.31 ± 342.09	392.96 ± 275.07	161.30 ± 167.24	77.27 ± 68.91	560.47 ± 288.37	34.32 ± 37.03	563.96 ± 464.11

The results were presented as the means ± SD.

**Table 2:** The PCA-X mode, OPLS-DA mode, and 999-permutation were used for multivariate exploration of clusters and trends among two groups of male and female in seven tissues.

Tissues	PCA-X		OPLS-DA		999-Permutation		Wilcoxon Test Differential/All
	R^2^X	Q^2^	R^2^Y	Q^2^	R^2^	Q^2^	
Heart	0.677	0.347	0.735	0.032	0.484	−0.321	2/55
Liver	0.917	0.767	0.871	0.717	0.845	−0.707	16/53
Spleen	0.594	0.259	0.999	0.897	0.928	−1.780	4/52
Lung	0.503	0.101	0.699	−0.072	0.703	−0.292	1/49
Kidney	0.720	0.279	0.946	0.579	0.844	−0.760	21/56
Brain	0.709	−0.210	0.529	−0.371	0.460	−0.360	0/42
Skeletal muscle	0.755	0.386	0.680	0.045	0.384	−0.371	8/57

Differential/all: differential is CL which has a significant difference, by Wilcoxon–Mann–Whitney test, in every tissue. All is all CL quantified in every tissue.

**Table 3:** Relative contents of CLs in different sex of liver and kidney.

**CLs**	**Liver**		***p***		**Kidney**		***p***
	**Male**	**Female**			**Male**	**Female**	
C66	0.50 ± 0.55	0.09 ± 0.09	0.011		0.53 ± 0.42	0.12 ± 0.07	0.013
C68	6.08 ± 4.90	0.68 ± 0.78	0.004		9.88 ± 7.22	2.64 ± 1.42	0.013
C70	62.04 ± 42.70	13.37 ± 12.73	0.011		12.17 ± 9.40	3.81 ± 2.23	0.018
C72	7.66 ± 4.63	1.82 ± 2.08	0.011		/	/	/
C74	/	/	/		0.21 ± 0.12	0.09 ± 0.04	0.018
C76	2.70 ± 2.27	0.48 ± 0.48	0.011		29.01 ± 20.10	10.67 ± 3.78	0.048
C78	0.05 ± 0.05	0.02 ± 0.01	0.011		1.14 ± 0.80	0.39 ± 0.12	0.048

The results were presented as the means ± SD. Wilcoxon–Mann–Whitney test was used to compare the sex difference between male and female.

## Data Availability

All data used during the study are available from the corresponding author by request.

## Supplementary Materials

The following are available online at https://www.mdpi.com/article/10.3390/molecules27206988/s1, Figure S1. The extract ion chromatograms at *m*/*z* 1449.9779 (No. 37) and its isotopic peak at *m*/*z* 1451.9902 in LC-QTOF-MS/MS analysis. Figure S2. PCA scores scatter plot of heart, liver, spleen, lung, kidney, brain, and skeletal muscle (R^2^ = 0.865, Q^2^ = 0.639). Figure S3. PCA scores scatter plot of heart, liver, spleen, lung, kidney and skeletal muscle (R^2^Y = 0.583, Q^2^ = 0.468). Figure S4. PCA scores scatter plot of liver and kidney based on males and females. Table S1. The detailed information of CLs obtained from seven tissues using UHPLC-QTOF-MS/MS. Table S2. The relative content distribution of CLs in heart, liver, spleen, lung, kidney, brain and skeletal muscle by LC-QTOF-MS/MS.

## Sample Availability

Samples of seven tissues are available from the authors.
