# Supplementary material for: Sex Differences of Cardiolipin in Tissue Distribution Based on Targeted Lipidomic Analysis by UHPLC-QTOF-MS/MS
Source: Molecules. 2022 Oct 18;27(20):6988. doi: 10.3390/molecules27206988 (PMC9612025; doi:10.3390/molecules27206988)
Supplement: Supplementary file 1 [file molecules-27-06988-s001.zip › molecules-1856561-supplementary.pdf]

Supplementary Materials

Sex Differences of Cardiolipin in Tissue Distribution Based on Targeted Lipidomic Analysis by UHPLC-QTOF-MS/MS

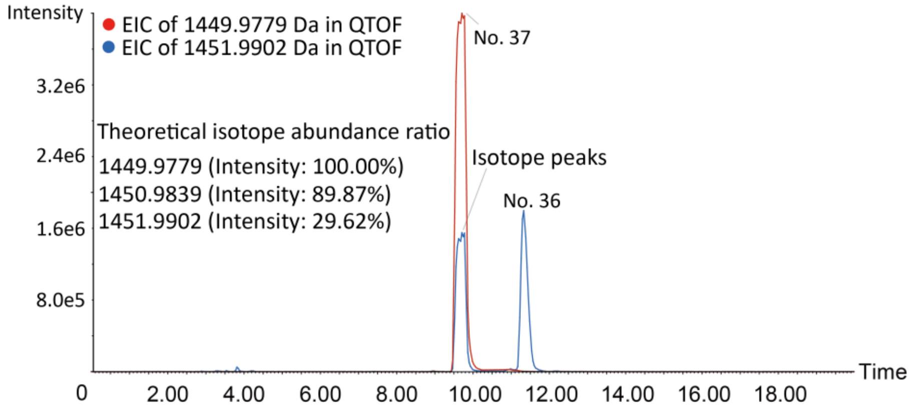

Figure S1. The extract ion chromatograms at m/z 1449.9779 (No.37) and its isotopic peak at m/z 1451.9902 in LC-QTOF-MS/MS analysis.

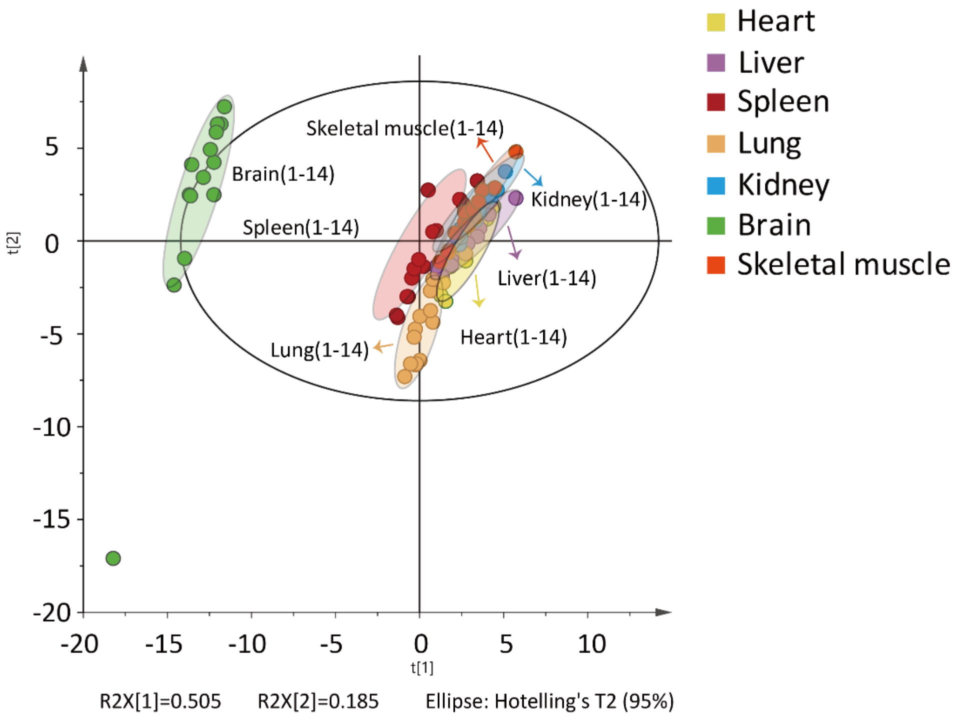

Figure S2. PCA scores scatter plot of heart, liver, spleen, lung, kidney, brain, and skeletal muscle ($R^2 = 0.865$, $Q^2 = 0.639$).

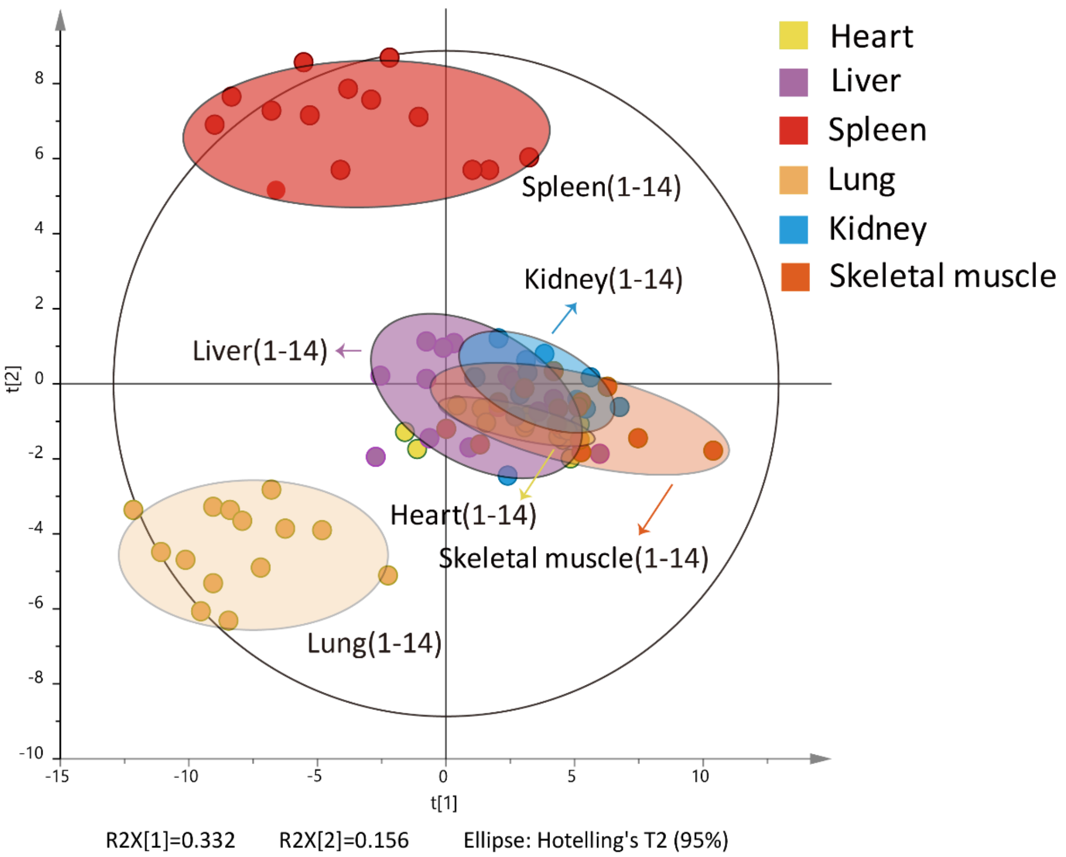

Figure S3. PCA scores scatter plot of heart, liver, spleen, lung, kidney and skeletal muscle ($R^2Y = 0.583$, $Q^2 = 0.468$).

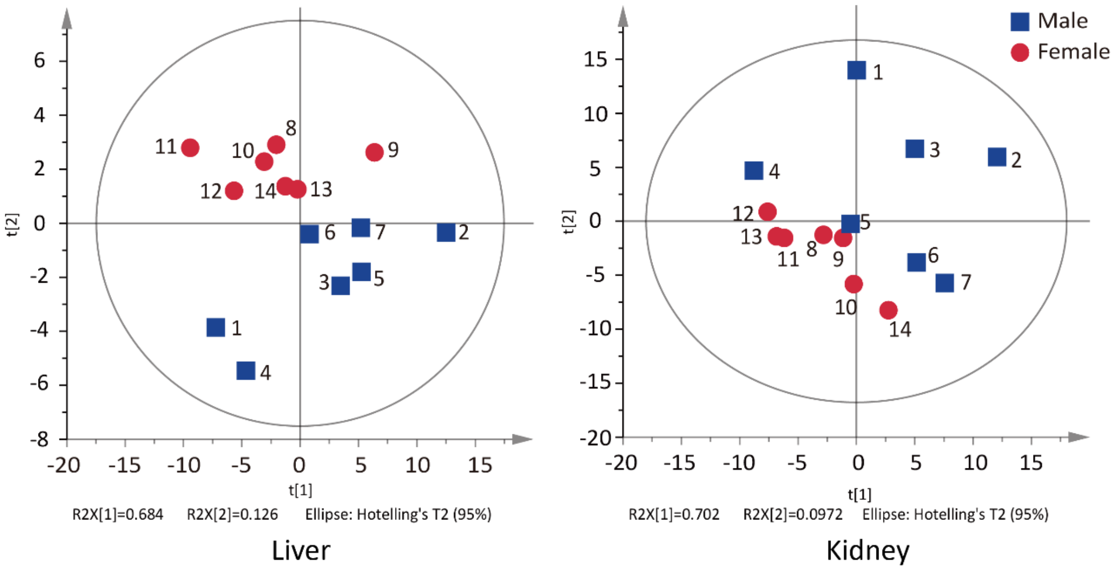

Figure S4. PCA scores scatter plot of liver and kidney based on males and females.

Table S1. The detailed information of CLs obtained from seven tissues using UHPLC-QTOF-MS/MS.

No.	Molecular species	Formula	$[M-H]^-$/exact	$[M-H]^-$/measure	Mass error (ppm)	R1+R2	R3+R4	Rt (min)
1	CL66:5	$C_{75}H_{136}O_{17}P_2$	1369.9175	1369.9188	1.0			8.70
2	CL68:2	$C_{77}H_{146}O_{17}P_2$	1403.9957	1403.9965	0.6	673.4800	673.4800	13.14
3	CL68:2	$C_{77}H_{146}O_{17}P_2$	1403.9957	1403.9965	0.6	671.4750	675.5020	13.16
4	CL68:3	$C_{77}H_{144}O_{17}P_2$	1401.9801	1401.9807	0.4	671.4670	673.4800	12.22
5	CL68:3	$C_{77}H_{144}O_{17}P_2$	1401.9801	1401.9807	0.4	699.5000	645.4630	11.91
6	CL68:4	$C_{77}H_{142}O_{17}P_2$	1399.9644	1399.9650	0.4	697.4800	645.4490	10.98
7	CL68:4	$C_{77}H_{142}O_{17}P_2$	1399.9644	1399.9650	0.4	671.4690	671.4690	11.38
8	CL68:5	$C_{77}H_{140}O_{17}P_2$	1397.9488	1397.9496	0.6	695.4700	645.4490	10.10
9	CL68:5	$C_{77}H_{140}O_{17}P_2$	1397.9488	1397.9496	0.6	697.4800	643.4370	10.14
10	CL68:5	$C_{77}H_{140}O_{17}P_2$	1397.9488	1397.9496	0.6	669.4490	671.4640	9.93
11	CL68:6	$C_{77}H_{138}O_{17}P_2$	1395.9331	1395.9369	2.7	669.4490	669.4490	9.13
12	CL68:6	$C_{77}H_{138}O_{17}P_2$	1395.9331	1395.9369	2.7	693.4530	645.4520	9.27
13	CL68:7	$C_{77}H_{136}O_{17}P_2$	1393.9180	1393.9180	0.0	695.4650	641.4230	8.42
14	CL68:7	$C_{77}H_{136}O_{17}P_2$	1393.9180	1393.9180	0.0	667.4430	669.4550	8.62
15	CL68:8	$C_{77}H_{134}O_{17}P_2$	1391.9018	1391.9091	5.2			7.91
16	CL70:3	$C_{79}H_{148}O_{17}P_2$	1430.0114	1430.0094	-1.4	699.4940	673.4920	13.24
17	CL70:4	$C_{79}H_{146}O_{17}P_2$	1427.9957	1427.9969	0.8	699.4930	671.4720	12.26
18	CL70:4	$C_{79}H_{146}O_{17}P_2$	1427.9957	1427.9969	0.8	697.4860	673.4720	12.24
19	CL70:5	$C_{79}H_{144}O_{17}P_2$	1425.9839	1425.9860	1.5	697.4810	671.4690	11.29
20	CL70:5	$C_{79}H_{144}O_{17}P_2$	1425.9839	1425.9860	1.5	695.4660	673.4760	11.02
21	CL70:6	$C_{79}H_{142}O_{17}P_2$	1423.9644	1423.9686	3.0	697.4810	669.4550	10.24

22	CL70:6	$C_{79}H_{142}O_{17}P_2$	1423.9644	1423.9686	3.0	695.4660	671.4590	10.48
23	CL70:6	$C_{79}H_{142}O_{17}P_2$	1423.9644	1423.9686	3.0	721.4830	645.4490	10.95
24	CL70:7	$C_{79}H_{140}O_{17}P_2$	1421.9488	1421.9526	2.7	669.4490	695.4660	9.43
25	CL70:7	$C_{79}H_{140}O_{17}P_2$	1421.9488	1421.9526	2.7	667.4350	697.4750	8.78
26	CL70:7	$C_{79}H_{140}O_{17}P_2$	1421.9488	1421.9526	2.7	693.4503	671.4692	9.86
27	CL70:8	$C_{79}H_{138}O_{17}P_2$	1419.9331	1419.9304	-1.9	693.4520	669.4470	8.76
28	CL70:8	$C_{79}H_{138}O_{17}P_2$	1419.9331	1419.9304	-1.9	667.4350	695.4660	8.74
29	CL70:8	$C_{79}H_{138}O_{17}P_2$	1419.9331	1419.9304	-1.9	645.4520	717.4520	8.93
30	CL70:8	$C_{79}H_{138}O_{17}P_2$	1419.9331	1419.9304	-1.9	669.4510	693.4530	9.05
31	CL70:9	$C_{79}H_{136}O_{17}P_2$	1417.9180	1417.9184	0.3	691.4310	669.4490	8.47
32	CL70:9	$C_{79}H_{136}O_{17}P_2$	1417.9180	1417.9184	0.3			8.12
33	CL72:4	$C_{81}H_{150}O_{17}P_2$	1456.0270	1456.0309	2.7	699.4990	699.4990	13.90
34	CL72:5	$C_{81}H_{148}O_{17}P_2$	1454.0114	1454.0117	0.2	697.4810	699.5000	12.31
35	CL72:6	$C_{81}H_{146}O_{17}P_2$	1451.9957	1451.9980	1.6	697.4810	697.4810	12.19
36	CL72:6	$C_{81}H_{146}O_{17}P_2$	1451.9957	1451.9980	1.6	721.4829	673.4869	11.40
37	CL72:7	$C_{81}H_{144}O_{17}P_2$	1449.9801	1449.9779	-1.5	695.4660	697.4810	9.73
38	CL72:7	$C_{81}H_{144}O_{17}P_2$	1449.9801	1449.9779	-1.5	721.4829	671.4692	11.23
39	CL72:8	$C_{81}H_{142}O_{17}P_2$	1447.9644	1447.9670	1.8	695.4660	751.4930	9.67
40	CL72:8	$C_{81}H_{142}O_{17}P_2$	1447.9644	1447.9670	1.8	693.4476	697.4758	10.17
41	CL72:9	$C_{81}H_{140}O_{17}P_2$	1445.9488	1445.9498	0.7	693.4530	695.4660	8.91
42	CL72:9	$C_{81}H_{140}O_{17}P_2$	1445.9488	1445.9498	0.7	643.4350	745.4830	9.29
43	CL72:9	$C_{81}H_{140}O_{17}P_2$	1445.9488	1445.9498	0.7	669.4387	719.4692	9.31
44	CL72:9	$C_{81}H_{140}O_{17}P_2$	1445.9488	1445.9498	0.7	671.4586	717.4462	9.62
45	CL72:10	$C_{81}H_{138}O_{17}P_2$	1443.9331	1443.9340	0.6	691.4330	695.4660	8.44
46	CL72:10	$C_{81}H_{138}O_{17}P_2$	1443.9331	1443.9340	0.6	693.4476	693.4476	8.95

47	CL72:10	$C_{81}H_{138}O_{17}P_2$	1443.9331	1443.9340	0.6			8.63
48	CL74:6	$C_{83}H_{150}O_{17}P_2$	1480.0270	1480.0249	-1.4	725.5080	697.4860	12.71
49	CL74:6	$C_{83}H_{150}O_{17}P_2$	1480.0270	1480.0249	-1.4	723.4980	699.4880	12.69
50	CL74:7	$C_{83}H_{148}O_{17}P_2$	1478.0114	1478.0170	3.8	723.5030	697.4890	11.64
51	CL74:7	$C_{83}H_{148}O_{17}P_2$	1478.0114	1478.0170	3.8	725.5140	695.4660	11.76
52	CL74:7	$C_{83}H_{148}O_{17}P_2$	1478.0114	1478.0170	3.8	721.4770	699.5000	12.29
53	CL74:8	$C_{83}H_{146}O_{17}P_2$	1475.9957	1475.9945	-0.8	723.4970	695.4660	10.83
54	CL74:8	$C_{83}H_{146}O_{17}P_2$	1475.9957	1475.9945	-0.8	719.4660	699.4940	11.42
55	CL74:8	$C_{83}H_{146}O_{17}P_2$	1475.9957	1475.9945	-0.8	721.4829	697.4812	11.33
56	CL74:9	$C_{83}H_{144}O_{17}P_2$	1473.9801	1473.9814	0.9	721.4830	695.4660	10.04
57	CL74:9	$C_{83}H_{144}O_{17}P_2$	1473.9801	1473.9814	0.9	719.4660	697.4812	10.50
58	CL74:9	$C_{83}H_{144}O_{17}P_2$	1473.9801	1473.9814	0.9	721.4829	695.4656	11.07
59	CL74:10	$C_{83}H_{142}O_{17}P_2$	1471.9644	1471.9697	3.6	745.4880	669.4440	9.33
60	CL74:10	$C_{83}H_{142}O_{17}P_2$	1471.9644	1471.9697	3.6	719.4660	695.4660	9.55
61	CL74:10	$C_{83}H_{142}O_{17}P_2$	1471.9644	1471.9697	3.6	721.4829	693.4476	10.02
62	CL74:11	$C_{83}H_{140}O_{17}P_2$	1469.9488	1469.9563	5.1	743.4670	669.4580	9.02
63	CL74:11	$C_{83}H_{140}O_{17}P_2$	1469.9488	1469.9563	5.1	719.4660	693.4480	9.16
64	CL76:7	$C_{85}H_{152}O_{17}P_2$	1506.0427	1506.0371	-3.7	723.4980	725.5200	12.97
65	CL76:7	$C_{85}H_{152}O_{17}P_2$	1506.0427	1506.0371	-3.7	699.4880	749.5150	13.19
66	CL76:8	$C_{85}H_{150}O_{17}P_2$	1504.0270	1504.0287	1.1	723.4920	723.4920	11.93
67	CL76:9	$C_{85}H_{148}O_{17}P_2$	1502.0114	1502.0123	0.6	749.5190	695.4660	11.16
68	CL76:9	$C_{85}H_{148}O_{17}P_2$	1502.0114	1502.0123	0.6	721.4770	723.4920	11.14
69	CL76:9	$C_{85}H_{148}O_{17}P_2$	1502.0114	1502.0123	0.6	745.4825	699.4889	11.95
70	CL76:10	$C_{85}H_{146}O_{17}P_2$	1499.9957	1499.9974	1.1	721.4830	721.4830	10.05
71	CL76:10	$C_{85}H_{146}O_{17}P_2$	1499.9957	1499.9974	1.1	747.4910	695.4660	10.40

72	CL76:11	$C_{85}H_{144}O_{17}P_2$	1497.9801	1497.9839	2.5	801.5080	695.4650	9.70
73	CL76:11	$C_{85}H_{144}O_{17}P_2$	1497.9801	1497.9839	2.5	721.4810	719.4660	10.07
74	CL76:11	$C_{85}H_{144}O_{17}P_2$	1497.9801	1497.9839	2.5	721.4659	719.4659	10.33
75	CL76:11	$C_{85}H_{144}O_{17}P_2$	1497.9801	1497.9839	2.5	745.4825	695.4656	10.79
76	CL76:12	$C_{85}H_{142}O_{17}P_2$	1495.9644	1495.9637	-0.5	743.4660	695.4660	9.36
77	CL76:12	$C_{85}H_{142}O_{17}P_2$	1495.9644	1495.9637	-0.5	769.4990	669.4490	9.07
78	CL76:12	$C_{85}H_{142}O_{17}P_2$	1495.9644	1495.9637	-0.5	719.4659	719.4659	9.47
79	CL76:12	$C_{85}H_{142}O_{17}P_2$	1495.9644	1495.9637	-0.5	745.4825	693.4476	9.83
80	CL76:13	$C_{85}H_{140}O_{17}P_2$	1493.9488	1493.9449	-2.6	743.4670	693.4470	8.69
81	CL76:13	$C_{85}H_{140}O_{17}P_2$	1493.9488	1493.9449	-2.6	719.4660	717.4520	8.90
82	CL78:10	$C_{87}H_{150}O_{17}P_2$	1528.0270	1528.0251	-1.2	749.5150	721.4780	11.41
83	CL78:11	$C_{87}H_{148}O_{17}P_2$	1526.0114	1526.0090	-1.6	749.5200	747.4920	10.71
84	CL78:11	$C_{87}H_{148}O_{17}P_2$	1526.0114	1526.0090	-1.6	745.4713	723.4863	11.45
85	CL78:12	$C_{87}H_{146}O_{17}P_2$	1523.9957	1523.9941	-1.1	719.4659	747.4908	10.45
86	CL78:12	$C_{87}H_{146}O_{17}P_2$	1523.9957	1523.9941	-1.1	721.4829	743.4713	10.93
87	CL78:12	$C_{87}H_{146}O_{17}P_2$	1523.9957	1523.9941	-1.1	699.4997	767.4677	10.92
88	CL78:13	$C_{87}H_{144}O_{17}P_2$	1521.9801	1521.9805	0.3	769.4884	695.4656	9.71

Table S2. The relative content distribution of CLs in heart, liver, spleen, lung, kidney, brain and skeletal muscle by LC-QTOF-MS/MS.

No.	Molecular species	Heart	Liver	Spleen	Lung	Kidney	Brain	Skeletal muscle
1	CL66:5	-1.48±0.33	-0.86±0.57	-1.55±0.52	-0.66±0.37	-0.71±0.45	/	-1.00±0.37
2	CL68:2	-1.76±0.58	-1.43±0.56	-1.64±0.50	/	-1.97±0.80	/	-1.56±0.49
4	CL68:3	-1.57±0.43	-1.08±0.44	-1.52±0.67	-2.52±0.53	-1.10±0.53	-2.07±0.51	-1.20±0.44
5	CL68:3	-2.54±0.45	-2.17±0.58	-2.12±0.83	-2.60±0.63	-2.24±0.39	-2.06±0.72	-1.85±0.53
6	CL68:4	-0.98±0.44	-0.63±0.54	-1.06±0.62	-1.09±0.41	-0.63±0.35	-1.39±0.88	-0.72±0.54
7	CL68:4	-1.64±0.37	-1.28±0.41	-1.47±0.50	-1.76±0.57	-0.83±0.50	-2.19±1.28	-1.42±0.42
8	CL68:5	-0.69±0.46	-0.46±0.46	-0.99±0.49	-0.94±0.37	-0.33±0.48	/	-0.54±0.49
10	CL68:5	-1.98±0.50	-1.10±0.65	-1.39±0.42	-0.57±0.36	-1.44±0.60	/	-1.55±0.43
11	CL68:6	-1.23±0.39	-0.18±0.85	-0.91±0.50	0.04±0.36	-0.59±0.35	/	-0.72±0.43
12	CL68:6	-0.02±0.38	-0.24±0.55	-0.75±0.50	-0.54±0.37	0.50±0.40	/	0.30±0.42
13	CL68:7	-1.50±0.33	-1.13±0.53	/	-1.22±0.36	-1.51±0.39	/	-1.27±0.47
14	CL68:7	-1.46±0.33	-1.74±0.40	/	-1.78±0.40	-1.43±0.53	/	-1.00±0.45
15	CL68:8	-1.50±0.32	/	/	/	/	/	-1.36±0.51
16	CL70:3	-2.64±0.59	-2.82±0.63	-2.32±1.05	-2.95±0.46	-2.66±0.70	-1.31±0.67	-1.79±0.59
17	CL70:4	-0.88±0.48	-0.51±0.46	-0.55±0.71	-1.51±0.39	-0.18±0.37	-0.69±0.67	-0.42±0.51
19	CL70:5	-0.21±0.41	0.07±0.44	-0.09±0.60	-0.78±0.39	0.57±0.32	-0.76±0.68	-0.10±0.44
20	CL70:5	-0.79±0.52	-0.16±0.63	-0.55±0.64	-0.68±0.34	-0.23±0.29	-1.37±0.67	-0.12±0.53
21	CL70:6	-0.13±0.38	0.61±0.52	0.02±0.56	0.22±0.37	0.51±0.31	-1.97±0.51	0.31±0.43
22	CL70:6	-0.62±0.34	-0.62±0.31	-0.37±0.42	-0.88±0.46	-0.03±0.49	/	-0.67±0.49
24	CL70:7	1.15±0.26	1.20±0.52	0.77±0.44	0.82±0.30	1.33±0.23	-1.92±0.45	1.24±0.31
25	CL70:7	-0.75±0.36	-1.34±0.58	-2.21±0.66	-2.14±0.58	-1.06±0.58	/	-0.53±0.55
28	CL70:8	-0.24±0.36	-0.76±0.56	-1.74±0.59	-1.16±0.49	-0.48±0.53	/	0.00±0.54
29	CL70:8	-0.96±0.39	-0.70±0.53	-1.27±0.56	-0.64±0.36	-0.17±0.48	/	-0.77±0.44
30	CL70:8	/	/	/	/	/	-1.33±0.49	/
31	CL70:9	-1.65±0.25	/	/	/	-1.94±0.38	/	-1.44±0.50
32	CL70:9	-1.20±0.29	-1.28±0.39	-1.80±0.41	-1.44±0.40	-0.97±0.40	/	-0.82±0.43
33	CL72:4	/	/	-1.89±0.86	/	-2.26±0.77	-0.22±0.78	-1.91±0.80
34	CL72:5	-0.88±0.50	-0.94±0.40	-0.57±0.71	-1.56±0.53	-0.15±0.32	-0.18±0.77	-0.20±0.45
35	CL72:6	1.01±0.45	1.15±0.36	0.76±0.59	0.17±0.39	1.50±0.24	-0.50±0.95	1.18±0.41
37	CL72:7	1.85±0.33	1.80±0.33	1.32±0.39	1.04±0.32	1.95±0.25	-1.17±0.62	1.76±0.36
39	CL72:8	2.67±0.23	2.05±0.36	1.59±0.35	1.30±0.28	2.29±0.22	-1.11±0.62	2.43±0.23

41	CL72:9	1.31±0.29	0.71±0.46	-0.47±0.43	-0.49±0.44	0.71±0.31	-1.38±0.78	1.09±0.35
42	CL72:9	0.37±0.29	0.42±0.55	0.02±0.44	0.20±0.29	0.87±0.23	-1.11±0.71	0.37±0.35
45	CL72:10	-1.15±0.29	-1.12±0.38	-2.55±0.78	-2.21±0.75	-0.99±0.39	/	-1.17±0.41
46	CL72:10	/	/	/	/	/	-0.78±0.67	/
48	CL74:6	-1.33±0.45	-1.47±0.38	-1.12±0.66	-1.96±0.66	-0.76±0.31	-0.88±0.69	-0.85±0.42
50	CL74:7	-1.19±0.38	-1.20±0.31	-1.25±0.39	-1.82±0.31	-0.91±0.21	/	-1.06±0.31
51	CL74:7	/	/	/	/	/	-0.90±0.61	/
52	CL74:7	/	/	/	/	/	0.04±0.82	-1.21±0.43
53	CL74:8	1.21±0.35	1.39±0.30	0.97±0.44	0.36±0.34	1.54±0.20	-1.86±0.74	1.42±0.31
54	CL74:8	-0.86±0.37	-0.78±0.44	-0.90±0.74	-1.43±0.37	-0.15±0.26	0.37±0.85	-0.68±0.36
56	CL74:9	1.37±0.35	1.31±0.31	0.90±0.38	0.45±0.33	1.35±0.22	-0.28±0.66	1.09±0.29
59	CL74:10	-1.06±0.34	-1.02±0.36	-1.52±0.65	/	-0.92±0.31	-0.44±0.72	-1.01±0.41
60	CL74:10	1.55±0.42	0.95±0.28	0.81±0.32	0.72±0.26	1.61±0.21	-0.93±0.60	0.89±0.46
62	CL74:11	0.34±0.27	-0.21±0.51	-1.08±0.56	-1.00±0.42	0.17±0.42	/	0.13±0.49
63	CL74:11	-0.12±0.51	-0.06±0.48	-1.16±0.68	-0.77±0.38	0.09±0.42	0.08±0.78	-0.15±0.41
64	CL76:7	-1.58±0.37	-2.46±0.39	-1.86±0.74	/	-1.79±0.46	/	-1.80±0.44
65	CL76:7	/	/	/	/	/	-1.63±0.67	/
66	CL76:8	-1.24±0.44	-0.92±0.45	-0.94±0.69	-1.96±0.58	-0.61±0.39	-1.17±0.68	-0.82±0.48
67	CL76:9	-0.30±0.51	0.05±0.34	-0.15±0.54	-0.61±0.28	0.16±0.23	-0.36±0.63	-0.22±0.44
70	CL76:10	0.28±0.55	-0.04±0.27	-0.17±0.39	-0.28±0.31	0.12±0.34	-0.63±0.59	0.16±0.39
71	CL76:10	-0.09±0.46	0.02±0.28	-0.30±0.48	-0.54±0.31	0.32±0.29	-0.77±0.75	-0.04±0.44
72	CL76:11	0.35±0.53	-0.49±0.39	-1.00±0.44	-1.08±0.30	0.17±0.40	/	0.35±0.44
73	CL76:11	0.89±0.37	0.71±0.28	0.03±0.49	-0.21±0.36	0.91±0.30	/	0.61±0.39
74	CL76:11	/	/	/	/	/	0.36±0.70	/
75	CL76:11	/	/	/	/	/	-0.36±0.48	/
76	CL76:12	-0.26±0.36	-0.15±0.60	-0.72±0.49	-0.15±0.33	0.18±0.36	-2.45±0.74	0.01±0.36
80	CL76:13	-0.77±0.47	-1.48±0.45	-2.38±0.30	/	-1.50±0.36	-1.99±0.38	-1.06±0.56
81	CL76:13	-1.26±0.43	-1.43±0.71	-1.83±0.80	-1.79±0.60	-0.74±0.44	-0.33±0.63	-1.34±0.50
82	CL78:10	-1.00±0.46	-2.10±0.59	-1.45±0.53	-1.71±0.39	-1.58±0.32	-0.70±0.73	-1.76±0.38
83	CL78:11	-1.21±0.47	-1.66±0.32	-1.52±0.44	-1.54±0.37	-1.06±0.37	/	-1.24±0.40
84	CL78:11	/	/	/	/	/	-0.59±0.65	/
85	CL78:12	-0.25±0.42	-0.38±0.25	-0.90±0.41	-0.97±0.27	-0.29±0.22	0.00±0.76	-0.39±0.32
88	CL78:13	-0.53±0.46	-0.31±0.25	-1.11±0.35	-1.15±0.30	-0.24±0.32	-0.44±0.43	-0.45±0.31

Quantitative results were obtained by dividing the peak area of each CL by the peak area of CL (14:0)4. For reducing the skewness in the distribution, the decadic logarithm (log10 transformation) of data was used. Results were expressed as the means ± SD.
